# Efficient Immuno-Modulation of T_H1_/T_H2_ Biomarkers in 2,4-Dinitrofluorobenzene-Induced Atopic Dermatitis: Nanocarrier-Mediated Transcutaneous Co-Delivery of Anti-Inflammatory and Antioxidant Drugs

**DOI:** 10.1371/journal.pone.0113143

**Published:** 2014-11-14

**Authors:** Zahid Hussain, Haliza Katas, Mohd Cairul Iqbal Mohd Amin, Endang Kumolosasi

**Affiliations:** Centre for Drug Delivery Research, Faculty of Pharmacy, Universiti Kebangsaan Malaysia, Kuala Lumpur, Malaysia; Mie University Graduate School of Medicine, Japan

## Abstract

The present study was conducted with the aim to investigate the immuno-modulatory and histological stabilization effects of nanocarrier–based transcutaneous co-delivery of hydrocortisone (HC) and hydroxytyrosol (HT). In this investigation, the clinical and pharmacological efficacies of nanoparticle (NP)-based formulation to alleviate 2,4-dinitrofluorobenzene (DNFB)-induced atopic dermatitis (AD) was explored by using an NC/Nga mouse model. *Ex vivo* visual examination of AD induction in experimental mice indicated remarkable control of NP-based formulations in reducing pathological severity of AD-like skin lesions. Therapeutic effectiveness of NP-based formulations was also evaluated by comparing skin thickness of AD-induced NP-treated mice (456±27 µm) with that of atopic mice (916±37 µm). Analysis of the immuno-spectrum of AD also revealed the dominance of NP-based formulations in restraining immunoglobulin-E (IgE), histamine, prostaglandin-E_2_ (PGE_2_), vascular endothelial growth factor-α (VEGF-α), and T-helper cells (T_H1_/T_H2_) producing cytokines in serum and skin biopsies of tested mice. These anti-AD data were further supported by histological findings that revealed alleviated pathological features, including collagen fiber deposition, fibroblasts infiltration, and fragmentation of elastic fibers in experimental mice. Thus, NP-mediated transcutaneous co-delivery of HC and HT can be considered as a promising therapy for managing immunological and histological spectra associated with AD.

## Introduction

Atopic dermatitis (AD) is chronically relapsing, non-contagious, and exudative; it typically manifests as pruritic dermatosis accompanied by perivascular infiltration of T-helper (T_H1_/T_H2_)-lymphocytes, mast cells, and immunoglobulin-E (IgE) [1], [2]. Common signs and symptoms of AD include the appearance of red to brownish-grey colored patches, severe itching, small raised bumps with exudates/transudates, and cracked/damaged stratum corneum (SC) [3], [4]. Genetic variability, environmental interactions, skin barrier disorders, and immunological reactions are among the proposed contributing factors [5], [6]; however, the exact pathogenesis of this allergic disorder is not well-established yet.

Mast cells and basophils are among the key effector cells in IgE-mediated allergic disorders, and play a key role in the pathogenesis of AD. These cells are stimulated in response to active cross-linking of AD-specific IgE with high affinity cell-surface IgE-receptors. On activation, these cells endure degranulation. Subsequently, they release active mediators, such as histamine, leukotrienes, and prostaglandin-E_2_ (PGE_2_) that play a critical underlying role in allergic reactions [7]. AD is further aggravated by the production of vascular endothelial growth factor-α (VEGF-α), a potent biomarker that induces hyperpermeability of blood vessels via abnormal neovascularization and endothelial cell proliferation. VEGF-α also acts as a chemoattractant for various inflammatory cells responsible for persistent aggravation in erythema and edema [7], [8]. In addition, release of numerous T_H1_/T_H2_-specific inflammatory mediators, such as interleukin (IL) types IL-4, IL-5, IL-6, IL-12p70, IL-13, interferon-γ (IFN-γ) and tumor necrosis factor-α (TNF-α) has been demonstrated in patients with AD [9], [10].

Topical glucocorticoids (TGs) are recognized as a well-established mainstay in relieving acute and chronic exacerbation of psoriasis and AD [11], [12]. The clinical significance of TGs in the prevention of these inflammatory disorders is concurrent with their vasoconstrictive, anti-inflammatory, immunosuppressive, and antiproliferative potency. However, long-term use of TGs is often accompanied by several local and systemic deleterious effects [13], [14] that limit clinical significance and exclude their application in chronic maintenance therapies. Hence, hydrocortisone (HC), a mildly potent agent of TGs, is administered percutaneously to minimize unwanted effects associated with use of TGs [3], [12]. In addition, HC is recognized as a mild agent due to its minimal systemic absorption compared to other TGs. This further improves its clinical applicability and therapeutic compliance [12]. To further broaden therapeutic feasibility and patient compliance, HC was coadministered with hydroxytyrosol (HT), a powerful oxygen free radical scavenger, skin soother, and wound healer.

Successful topical/percutaneous delivery of drugs has been limited due to the penetration barriers provided by the SC [15]. Various active and passive penetration-enhancing approaches, including chemical enhancers [16], electroporation [17], micro-needles [18], and several vesicular delivery systems such as colloidal carriers [19], liposomes [20], ethosomes [21], solid lipid nanoparticles [22] and nano-emulsions [23] have been investigated to overcome this problem. Besides, polymeric nanoparticles (NPs) are well recognized as an advanced non-invasive technique to facilitate delivery of therapeutics into the skin [24] without detrimental effect on SC [25], [26]. The usefulness of polymeric NPs has also been highlighted by Hussain and co-workers in achieving therapeutic dose in the epidermis and dermis and to reduce systemic absorption of TGs and thus minimizing their side effects [3]. Moreover, the HC-loaded polymeric NPs were more efficient in alleviating the signs and symptoms of dermatosis in mice compared to HC cream of equivalent and higher concentrations [27]. The successfulness of NP-based delivery has been associated with their nano-range size and excellent bio-pharmaceutical properties, such as high entrapment efficiency (EE), controlled release rates and insignificant enzymatic degradation. Among various biodegradable and biocompatible polymers used for preparing NPs, chitosan (CS) has generated much enthusiasm due to its mucoadhesive and transepidermal penetrative properties via regulation of intercellular tight junctions [28].

The aim of this investigation was to explore the anti-AD effect of HC/HT co-loaded NP-based formulation in terms of its modulatory effects on the immuno-spectrum of T_H1_/T_H2_ specific cytokines. In the present study, AD was induced in NC/Nga mice by applying 2,4-dinitrofluorobenzene (DNFB). Mice were treated with the test formulations and blood samples were collected for immunological analysis. Moreover, the dorsal skin of AD-induced mice was surgically excised to perform immunohistochemistry (IHC) on infiltrated biomarkers responsible for AD. Clinical data were further harmonized by conducting several histological examinations to assess histopathological features of skin in AD-induced mice including, intensity of collagen fibers deposition, thickening/fragmentation of elastic fibers, and skin fibrosis.

## Materials and Methods

### Materials

Eight-week-old NC/Nga mice were purchased from RIKEN BioResource Center, Japan. Isoflurane was obtained from Piramal Healthcare Limited (Kuala Lumpur, Malaysia). DNFB, acetone, CS (MW, 70 kDa; deacetylation degree, 85%), HC (base form), and HT were purchased from Sigma Aldrich Chemicals Co. Ltd. (Kuala Lumpur, Malaysia). Halt protease inhibitor cocktail and cell lysis buffers were sourced from Thermo Scientific (Kuala Lumpur, Malaysia). The chemicals used to conduct immunological studies included IgE enzyme linked immunosorbent assay (ELISA) kit (Abcam Chemical, Malaysia), PGE_2_ ELISA kit (Cayman Chemical, Malaysia), histamine ELISA kit (Abnova Chemicals, Malaysia), VEGF-α ELISA kit (Life Technologies, Malaysia), and multi-analyte profiling Procarta assay kit (Affymetrix, Malaysia). All other chemicals were of analytical grade and sourced from research laboratories of Universiti Kebangsaan Malaysia.

### Preparation of HC/HT co-loaded NPs

The HC/HT co-loaded NPs with optimized physicochemical characteristics were prepared according to Hussain et al. [3]. A volume of 25 mL of CS solution (0.2% w/v, prepared in 1% v/v acetic acid, pH 5.0) was incubated with HC and HT (1 mg/mL of each in 30∶70 ratio of ethanol/water) for 30 min. Co-loaded NPs were spontaneously formed by adding 10 mL of pentasodium tripolyphosphate (TPP) solution (0.1% w/v, dissolved in distilled water) dropwise under constant magnetic stirring (700 rpm). The resulting NPs were harvested by ultracentrifugation (28,000 rpm) for 30 min using an Optima L-100 XP Ultracentrifuge with an NV 70.1 Ti rotor (Beckman-Coulter, USA). Pellets of co-loaded NPs were subsequently lyophilized (Scanvac CoolSafe, Chemoscience, Thailand) at −40°C for 24 h.

### Physicochemical characterization of prepared HC/HT co-loaded NPs

Co-loaded NPs recovered after ultracentrifugation were resuspended in 3 mL distilled water prior to measurement of mean particle size, polydispersity index, and zeta potential using an ZS-90 Zetasizer (Malvern Instruments, UK). All measurements were performed in triplicate at 25°C with a detection angle of 90°. Data are reported as mean ± standard deviation (S.D.).

Percent of EE (%EE) and loading capacities (%LC) of both loaded drugs were determined using high performance liquid chromatography (HPLC). Firstly, the corresponding calibration curves were made by subjecting a range of standard solutions (1 to 1000 µg/mL) of HC and HT to HPLC analysis (HPLC system with Waters 600 controller, in-line degasser AF, 2707 Autosampler, and 2998 Photodiode Array Detector, and Waters symmetry C_18_ column [250×4.5 mm; 5 µm]). The mobile phase for the elution of HC and HT consisted of methanol, acetonitrile, and water at a ratio of 15∶27∶58 (v/v) and was delivered at a flow rate of 1 mL/min with an injection volume of 20 µL. The maximum wavelength (λ_max_) used to measure HC and HT was 248 nm and 280 nm, respectively. %EE and %LC of both loaded drugs were calculated in accordance to equations 1 and 2, respectively [29].
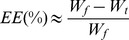
(Equation 1)

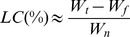
(Equation 2)Where, *W_t_* is the total initial amount of HC or HT, and *W_f_* is the amount of free drug in the supernatant after ultracentrifugation. *W_n_* is the average weight of lyophilized drug-loaded NPs. All measurements were performed in triplicate and the data are reported as mean ± S.D.

### Compounding of NP-based formulations

The prepared HC/HT co-loaded NPs were compounded as topical cream formulations. Two over-the-counter creams, QV and aqueous cream were used as vehicle bases. QV-cream is composed of higher contents of light liquid paraffin (85% w/w), white soft paraffin (15% w/w) and glycerol (10% w/w) than the aqueous cream (liquid paraffin 6.0% w/w and white soft paraffin 15% w/w). In addition, QV-cream contains natural oils such as squalene and other lipids (stearic acid, glycerol strearate, and stearyl alcohol) which improve occlusiveness and bioadhesion of the cream on the inflamed skin. To achieve adequate uniformity of contents and to attain homogenous dispersive systems, vehicle bases were liquefied in water bath (BW-20G, Lab. Companion, USA) at 50°C. With subsequent vigorous blending of HC/HT co-loaded NPs into QV- and aqueous-vehicle bases, the NP-based formulations, Q-HC-HT-NPs and A-HC-HT-NPs respectively, were compounded.

For that, 1562 mg of lyophilized HC/HT co-loaded NPs was blended into100 g of either QV or aqueous vehicle bases. The incorporated amount of lyophilized co-loaded NPs was calculated from the formula of %LC (in equation 2) that was equivalent to commercial 0.5% HC cream (DermAid 0.5%) used as positive control in this investigation. Accordingly, the compounded QV- and aqueous-NP–based formulations contained HC equivalent to 0.5% and HT of 0.42% w/w. Moreover, non-NPs–based formulations were compounded in a similar way to achieve an equivalent percentage of HC and HT as that of NP-based formulations. Briefly, 500 mg HC and 420 mg HT were homogenized into 100 g of each preliquefied QV- and aqueous-vehicle creams to produce non-NPs–based formulations (Q-HC-HT-cream and A-HC-HT-cream). The compounded formulations were then placed in amber glass containers and stored in a cool and dry place prior to further analysis.

### Characterization of NPs- and non-NP-based formulations

NP- and non-NP–based test formulations were characterized for uniformity of drug content, rheological behavior, pH, and apparent viscosities.

### Determination of drug contents

In this study, standard calibration curves were generated by subjecting various HC and HT standards (1 to 1000 µg/mL) to HPLC analysis. Each test formulation (1 g) was placed in a separate volumetric flask prefilled with 60 mL of solvent mixture (with 90∶10 v/v ratio of methanol and acetonitrile), and the volume of each flask was made up to 100 mL using the same solvent mixture. Volumetric flasks were then shaken overnight using a hot plate stirrer (37±0.5°C) for complete extraction of drugs either from non-NPs–based or NP-based formulations. The extracted mixtures were left undisturbed. Then, mixtures were passed through a 0.45-µm polytetrafluoroethylene filter separately followed by subsequent 10-fold dilution of each extracted filtrate using the same solvent mixture. Diluted samples were analyzed by HPLC; the peaks and area under the curve were subjected to regression analysis for drug quantification.

### Rheological characterization

Flow mechanics and apparent viscosities of QV- and aqueous- non-NPs–based and NP-based formulations were studied using a Bohlin Gemini Rheometer and Viscometer (Aimil Ltd. Instrumentation & Technology). The rheometer was engaged with a cone and plate system (1°/40 mm) and fully integrated Peltier device—a forced gas oven with optional liquid nitrogen cooling and electrical heating facilities. Applied strain rates ranged from 0.005 to 300 s^−1^ with broad torque range. Each experiment was run for 2 min, followed by 2 min at a constant strain rate of zero. All measurements were performed in triplicate at 32°C with accurately controlled shear rates.

### Measurement of pH

The pH of test formulations was determined with an FE20-FiveEasy pH meter (Mettler Toledo, USA). Prior to analysis, test formulations were kept at room temperature (22±2°C) for 30 min. The pH meter probe was carefully immersed into each cream while avoiding contact with the base of the container. To enhance the accuracy of experiment, the cream was thoroughly stirred with the probe and an equilibration period of 1 min applied. The probe was washed thoroughly with 10% ethanol and then distilled water before subsequent experiments. Each experiment was performed in triplicate and data reported as mean ± S.D.

### *In vivo* animal studies using an NC/Nga mouse model

#### Experimental animals

*In vivo* animal studies were performed using 8-week-old single-line NC/Nga mice obtained after serial breeding to reduce genomic diversity. Mice were acclimated for one week in Individually Ventilated Cage (IVC) assemblies with inlet air filters, and kept in an air-conditioned environment with 12-h light/12-h dark cycle at a well-controlled temperature (22±1°C) and humidity (60%±5%). Mice were provided with laboratory diet and water *ad libitum*. Experimental protocols for animal handling were in accordance with the National Institute of Health (NIH) guidelines and approved by the Animal Ethics Committee of Universiti Kebangsaan Malaysia (UKMAEC) under the project approval code (FF/2011/HALIZA/30-NOVEMBER/408-NOVEMBER-2011-DECEMBER-2012).

### Protocol for the induction of AD and treatment groups

At the end of the acclimation period, mice were shaved in the dorsal body region taking extreme precaution to avoid any skin abrasion. AD induction was initiated by sensitizing anesthetized mice with 100 µL of 0.15% solution of DNFB in acetone/olive oil (3∶1) applied onto the shaved dorsal skin once on days 1 and 5. To enhance the AD-inducing efficiency of DNFB and to avoid counter plaster effects of skin sebum, barrier disruption was achieved by treating the shaved dorsal skin with 150 µL of 4% sodium dodecyl sulfate 3 h prior to applying DNFB. On days 9, 11, and 13, 100 µL of 0.2% DNFB was reapplied to sensitized mouse dorsal skin as described previously. NC/Nga mice were then randomly divided into 9 groups (n = 6 per group). Normal mice (NRM) were considered as the baseline group and used to evaluate normal anatomical and immunological parameters. The second group was used as the negative control (NG-CONT); containing mice received repeated topical DNFB applications without pharmacological treatment. The third and fourth groups were vehicle groups (VGRs) consisting of AD-induced mice (DNFB) treated with vehicle creams (QV and aqueous creams), respectively. The fifth group consisted of AD-induced mice treated with commercial DermAid 0.5% cream and used as the positive control (POS-CONT) group. The sixth (Q-HC-HT-cream) and seventh (A-HC-HT-cream) groups consisted of AD-induced NC/Nga mice treated with QV- and aqueous-based non-NPs formulations, respectively. Similarly, the eighth and ninth groups were AD-induced mice treated with QV- and aqueous-based NP-based co-loaded formulations, Q-HC-HT-NPs and A-HC-HT-NPs, respectively. Following AD induction, mice were treated for 6 weeks with continuous challenge of 0.2% DNFB during the course of treatment.

### Euthanization of experimental animals: Collection of serum and skin tissues

At the end of treatment period, all the experimental animals were subjected to euthanization by isoflurane and the blood and skin samples were collected for subsequent immunological and histological examinations.

### Separation of serum from collected blood samples

Blood samples, withdrawn by cardiac puncture, were individually placed into 2-mL pre-labeled Eppendorf tubes. The tubes were left undisturbed for 30 min at 25±1°C to accelerate clot formation. Subsequently, all samples were incubated at 4°C overnight to contract the formed blood clots. Biological samples were then subjected to centrifugation (2,000 *g*) at 4°C for 15 min. Serum (pale yellow liquid) that settled on top of each centrifuged tube was carefully withdrawn by micropipette and placed into another pre-labeled Eppendorf tube, and stored at −80°C until further analysis.

### Collection of skin samples for histological analysis and IHC

Dorsal skin samples were surgically excised from AD-lesional sites of all NC/Nga mice. Collected skin samples were cleaned with isopropyl alcohol and stored in 10% buffered formalin for histological analysis. In addition, surgically excised skin samples were wrapped in aluminum foil and stored at −80°C for subsequent IHC analysis.

Prior to performing IHC analysis, endogenous and exogenous AD-responsible mediators infiltrated into lesional skin tissues were extracted by making skin homogenates from surgically excised skin. To achieve this, 1 g of excised skin tissue was placed in a 2-mL plastic tube prefilled with 3 grinding iron beads (2.8 mm in diameter). Then, 300 µL of ice-cold cell lysis buffer was added to each tube as extraction and biological media. The extraction tubes were then homogenized 3 times using a tissue homogenizer (Bioprep-24 homogenizer) with a preset speed (6 m/s) of 40 s with subsequent resting period of 20 s for each extraction cycle. At the end of tissue homogenization, the extraction tubes were placed onto ice block to prevent proteolytic degradation of extracted and solubilized proteins. Furthermore, halt protease inhibitor cocktail was also added into each extraction tube prior to perform homogenization of excised skin tissues. The halt protease inhibitor cocktail effectively blocks various proteases (serine, cysteine, aspartic acid, and aminopeptidases) that typically present in cellular/tissue homogenates. Extraction tubes were then centrifuged (13,000 rpm) for 2 min and tissue homogenate (whitish liquid) that settled on top was carefully removed and stored at −80°C for IHC analysis.

### *In vivo* immunological studies

In this study, IgE, histamine, PGE_2_, VEGF-α, and AD-responsible T_H1_ and T_H2_ specific cytokines, such as IL-4, IL-5, IL-6, IL-12p70, IL-13, IFN-γ, and TNF-α were assessed in the serum and skin tissue homogenates of all experimental animals. Data are expressed as mean ± SD.

### ELISA assay

Expression levels of IgE (IgE ELISA kit; Cat. No. KA 1944, Abnova Chemicals), histamine (histamine EIA ELISA kit; Cat. No. KA 0258, Abnova Chemicals), PGE_2_ (PGE_2_ Express EIA kit; Cat. No. 500141, Cayman Chemicals), and VEGF-α (VEGF-α ELISA kit; Cat. No. KA 0258, Abnova Chemicals) were measured in serum and skin homogenates by specific sandwiched-type ELISA according to the respective manufacturer's instructions.

### Procarta immunoassay

The expression intensity of major exogenous/endogenous AD-responsible cytokines (i.e.; IL-4, IL-5, IL-6, IL-12p70, IL-13, IFN-γ, and TNF-α) in serum and skin homogenates were determined using Procarta immunoassay. Procarta is a high-throughput multiplex immunoassay with higher reproducibility and enables the simultaneous quantification of multiple protein targets. Furthermore, it is a highly sensitive assay (>5 pg/mL) and can effectively multiplex several inflammatory mediators in a sample unit.

### Histological examinations

Dorsal skin specimens (5 mm) obtained after euthanization of NC/Nga mice were punched by skin biopsy needle and fixed in 10% buffered formalin. Skin specimens were then processed by a series of solvents, embedded in paraffin wax, and serially sectioned (5 µm) using a microtome. Sections were affixed to glass sample slides by the fishing method. Slides were then rehydrated and dehydrated by bathing them in various concentrations of alcohol (95%, 80%, 70%, and 50% v/v). Then, slides were stained with hematoxylin-eosin (H & E) and Masson's trichrome stains to observe histopathological features of the skin and to examine variable deposition of collagen fibers (green color) and skin fibrosis at lesional skin sites, respectively. The sectioned skin specimens were also stained with Verhoeff-Van Giesen (VVG) stain to examine pathological changes, such as atrophy, thickening, and fragmentation of elastic tissue fibers. Finally, stained skin specimens were examined for various pathological changes in skin infrastructure, collagen fibers, and elastic fibers under a light microscope with image analysis software (VideoTesT-Master Morphology: Video TesT, St Petersburg, Russia).

### Statistical analysis

Data are presented as mean ± S.D., and analyzed using either paired t-tests or analysis of variance (ANOVA) followed by Tukey's post-hoc analysis. For contents uniformity, pH values, apparent viscosities, and rheological data, differences among the groups were considered statistically significant when p<0.05. For immunological testing, **p<0.005 indicated a significant difference between NP-based formulations and NG-CONT/VGRs groups. Similarly, **^##^**p<0.005 indicated a significant difference between the NRM and NG-CONT/VGRs groups.

## Results and Discussion

### HC/HT co-loaded NPs with optimal physicochemical characteristics

The optimized co-loaded NPs prepared in this study had a mean particle size of 244±21 nm, with zeta potential of +38±4 mV. The %EE of these co-loaded NPs was measured to be 79±7 and 59±3 for HC and HT with %LC of 32±4 and 27±3 for HC and HT, respectively. Furthermore, the in-vitro drug release of HC/HT co-loaded CS NPs conducted at pH 4.0 (intact skin) and 7.4 (inflammatory skin lesion) demonstrated that the co-loaded CS NPs exhibited biphasic release pattern with the initial fast release up to 12 h and subsequent slow release up to 24 h (Fig. S1). The higher pH (7.4) also favors the release of drugs (Figure S1). This could be explained on the basis that at higher pH value, the positively charged amino groups (−NH3+) of CS NPs might be converted into unionized form (−NH2). As a result, the ionic cross-linking extent between CS and TPP might be reduced and caused loosening of CS NPs matrices [30] and facilitating release of the loaded drugs. On the other hand, in an attempt to assess clinical significance of NPs-system in alleviating AD-like skin lesions in NC/Nga mice, the co-loaded NPs were compounded into QV- and aqueous-vehicle bases.

### Characterization of NP- and non-NP–based topical formulations

#### Drug contents

Drug contents determination was carried out to ensure homogeneous dispersion of entrapped drugs in NP-based and non-NP-based formulations. The absolute recovery of HC obtained from QV- and aqueous-based co-loaded NP-based formulations was measured to be ∼76.2% w/w and ∼74.7% w/w, respectively. On the other hand, the absolute recovery of HT was ∼75.5% w/w and ∼73.9% w/w from Q-HC-HT-NPs and A-HC-HT-NPs, respectively. These findings clearly showed that the recovery of drugs was more efficient from QV-based than aqueous-based formulation. This could be explained by solvent–solute interaction between the extracting solvent system and QV- or aqueous-cream matrices that might affect the ability of the extracting solvent to dissolve the cream ingredients prior to drug release. In contrast, non-NPs–based formulations (i.e., Q-HC-HT-cream & A-HC-HT-cream) had shown a significant higher percentage of absolute recovery of both drugs (p<0.05, paired t-test). The recovery of HC and HT from Q-HC-HT-cream was ∼91.8% w/w and ∼90.1% w/w, respectively. Whereas, the recovery of HC and HT from A-HC-HT-cream was ∼87.4%w/w and ∼92.7% w/w, respectively. The lower absolute recovery of entrapped drugs from NP-based formulations was an expected finding, and is due to incomplete extraction because some drugs might remain entrapped inside the NPs matrices. The intactness of the CS polymeric network might prevent the entrapped drugs from being solubilized and released into the extracting solvent system. On the other hand, the absence of a polymeric network in non-NPs–based systems enables direct release of both entrapped drugs from the cream matrices into solution form. Furthermore, ex-vivo Franz diffusional drug permeation study revealed that NPs-based formulations significantly reduced the permeation, permeation flux (J/h), and permeability coefficient (Kp) of HC and HT across the dermatomed mouse skin (S2). This could be explained by the fact that CS and cream matrix provided extra barriers for drug diffusion and permeation across mouse skin. Besides, CS NPs could attach to the skin appendages and skin tissues due to mucoadhesive property of CS and thus, their active contents could be released locally. This therefore could reduce the risk-benefit ratios of TGs.

### Rheological behavior

**Figure 1:**
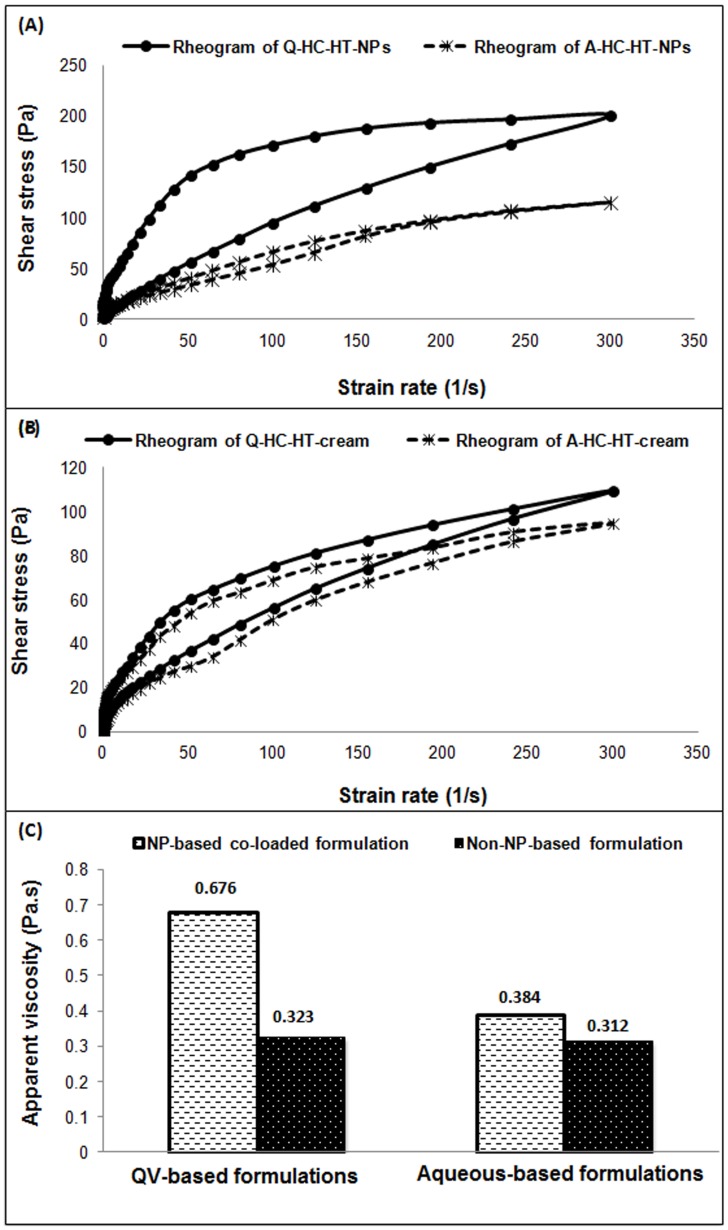
Flow characteristics of NP- and non-NP-based formulations. Rheogram of QV and aqueous NP-based formulations (A), Rheogram of QV and aqueous non-NPs–based formulations (B), apparent viscosities of NP- and non-NP–based formulations (C). Results represent the non-Newtonian mechanics of NPs- and non-NPs-based formulations. NPs, nanoparticles.

Rheological property is an imperative parameter in the comprehension of flow characteristics and colloidal stability of formulations [31]. Rheograms of QV- and aqueous-based non-NP- and NP-based formulations are shown in Fig. 1. The rate and extent of shear stress on the QV- and aqueous-based NP-based formulations were proportionally dependent on the applied strain rates (Fig. 1 [A]). Furthermore, they demonstrated pseudoplastic flow. These results are in accordance with a previous study [32], which described that the rate and extent of shear stress of any formulation proportionally correlated with the applied strain rate would follow non-Newtonian mechanics. Furthermore, the QV-based co-loaded NPs-based formulation was observed to be more thixotropic in nature compared to the aqueous-based formulation. Thixotropy and viscosity greatly influence release rate of drugs from the cream matrices, occlusiveness and bio-adhesion of creams when they are applied onto the skin [33]. Higher thixotropy and viscosity improve adhesiveness of a cream for a longer period of time and thus, enhance its efficacy [33]. In present study, QV-cream had shown slightly higher thixotropy and viscosity compared to the aqueous cream that might also increase intimate contact between the release NPs and the skin that led to higher anti-AD efficacy of QV-based NPs formulations compared with aqueous-based ones. Besides, the viscosity and thixotropy of topical cream influence the spreadability and permeation of drugs into the epidermis and dermis [34]. On the other hand, the non-NPs-based formulations had also demonstrated non-Newtonian mechanics with similar flow characteristics between QV- and aqueous-based systems (Fig. 1 [B]). Moreover, QV- and aqueous-based co-loaded NPs-based formulations were more found to be more thixotropic compared to non-NPs-based formulations. The authors described that the increase in shear stress observed with increasing of strain rate was expected to be due to structural breakdown of weak bonds that hold particulate matters together [35]. This might lead to the formation of aggregates and reduce apparent viscosity.

On the other hand, Q-HC-HT-NPs had also shown higher apparent viscosity (0.676±0.022 Pa.s) compared to A-HC-HT-NPs as shown in Fig. 1 [C]. This could be due to the thickening effect of paraffinum subliquidum (85% w/w) in the QV-based formulation. Moreover, higher value of apparent viscosity in case of QV-based formulation (0.323±0.012 Pa.s) was also observed for non-NPs-based compared to aqueous-based non-NPs-based formulation (0.312±0.09 Pa.s). The authors of the present study demonstrated that higher apparent viscosity observed in case of NPs-based formulation is expected to be due to the lyophilized NPs in NPs-based formulation that might tend to reduce shear thinning effects on contained formulation [36].

### Measurement of pH

This investigation exhibit that the pH of QV-based co-loaded NPs-based formulation (5.72±0.17) was slightly higher than aqueous-based co-loaded NPs-based formulation (5.61±0.19). These values of pH were significantly lower than non-NPs-based formulations, which were measured as pH 6.23±0.07 and 6.02±0.11 for Q-HC-HT-NPs and A-HC-HT-NPs, respectively. The authors of the present study anticipated that the presence of the intact polymeric form of CS (pKa, ∼6.5) or its acidified form might be the reason for lower pH of NP-based formulations.

### *In vivo* clinical efficacy

#### Severity of dermatosis

**Figure 2:**
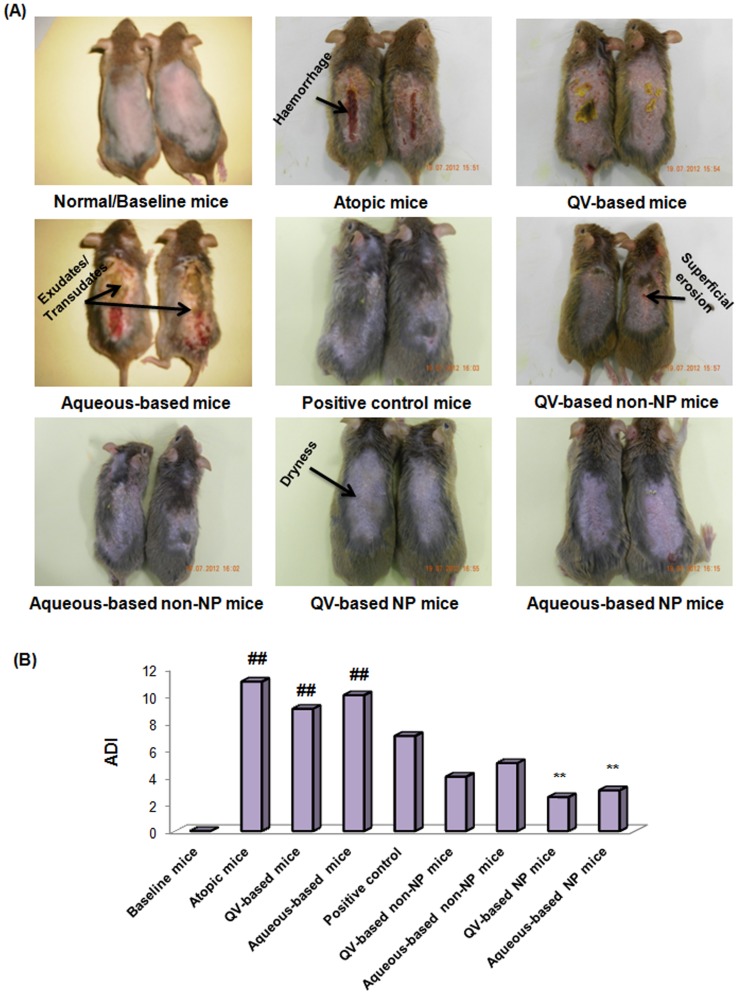
Clinical effectiveness of NP-based formulations compared to other groups tested. Digital images (A) and ADI (B) of untreated and treated AD-induced NC/Nga mice. Digital images represent the severity of AD symptoms at the end of treatment in term of ADI. AD, atopic dermatitis; ADI, atopic dermatitis index/scores; NP, nanoparticles. **Digital images were taken from the same animals as previously reported in Hussain et al. [3].

Therapeutic effectiveness of NP-based formulations using an NC/Nga mouse model was explored by examining its representative symptom, dermatosis severity. Severity of AD was assessed by 2 dermatologists blind to groups tested according to the criteria described previously by Park et al. [37]. The severity of AD was evaluated as AD index/score (ADI) established according to the following criteria: (1) erythema/hemorrhage, (2) dryness/scaling, (3) edema/swelling, and (4) erosion/excoriation, each of which were scored as 0 (none), 1 (mild), 2 (moderate), or 3 (severe). The sum of the individual scores was then taken as the ADI. Fig. 2 addressed that AD-induced mice that had not received any treatment (NG-CONT) had shown highest severity of dermatosis (ADI, 10.5). The obtained image of NG-CONT mice had shown various pathological features such as severe erythema, hemorrhage, edema, release of exudates/transudates, superficial erosion, deep excoriation, intense itching, and dry skin (Fig. 2 [A]). The obtained digital photographs also highlighted that the severity of dermatosis in VGR groups (Q-VGR and A-VGR) was similar to the NG-CONT group, but with reduced hemorrhage, edema, and erythema. In contrast, AD-induced mice treated with commercial DermAid 0.5% formulation (POS-CONT) had better control of AD symptoms (ADI, 7.0). On the other hand, NP-based formulations demonstrated remarkable control of AD symptoms compared to non-NPs–based formulations. The ADI of Q-HC-HT-NPs and A-HC-HT-NPs were significantly lower than Q-HC-HT-cream and A-HC-HT-cream as shown Fig. 2 [B]. Furthermore, QV-based NPs formulation was more effective in controlling the severity of dermatosis compared with aqueous-based NPs formulation. This finding could be related to the higher drug permeation flux across the NC/Nga mouse skin when the drugs were incorporated into QV-cream (S2). Higher contents of glycerol, light liquid paraffin and white soft paraffin in QV-cream compared to aqueous cream higher might attribute to higher drug permeation flux. QV-cream also provides better skin hydration that facilitates drug permeation across the skin. Besides, natural oil such as squalene, stearic acid and stearyl alcohol could further improve drug permeation by improving adhesiveness of QV-cream on the skin. Therefore, these findings suggested that NP-based formulations were more effective in maintaining skin integrity during the course of dermatosis and treatment, and were associated with minimal symptoms of dryness and erythema.

### Thickness of excised dorsal mouse skin

At the end of the 6-week treatment course, the anti-AD potential of test formulations was evaluated by measuring the thickness of excised dorsal skin of NC/Nga mice. NG-CONT mice had a significant increase in the thickness of dorsal body skin (916±37 µm) compared to normal/baseline mice (412±19 µm; p<0.005, one-way ANOVA). The increased skin thickness observed in NG-CONT mice was expected to be caused by activation of underlying inflammatory cascades associated with AD pathogenesis. These inflammatory reactions might provoke various pathological processes, such as accumulation of inflammatory mediators in papillary/reticular layers of dermis, neovascularization, keratinization, and epithelization. Likewise, the skin thickness of Q-VGR and A-VGR mice was 822±41 and 842±31 µm, respectively. Contrary to that, commercial DermAid 0.5% reduced skin thickness by ∼30% compared with the NG-CONT group. It was also revealed that NP-based formulations were superior in maintaining the thickness of AD-induced skin as skin thickness was reported as 456±27 and 476±24 µm for Q-HC-HT-NPs and A-HC-HT-NPs, respectively. Skin thickness of mice treated with QV- and aqueous-based non-NPs formulations was 590±27 and 612±27 µm, respectively. The lower skin thickness observed in mice treated with NP-based formulations was expected to be due to the efficient delivery of HC and HT into the epidermis and dermis by CS NPs.

### *In vivo* immunomodulatory efficacy

#### Expression of IgE

**Figure 3:**
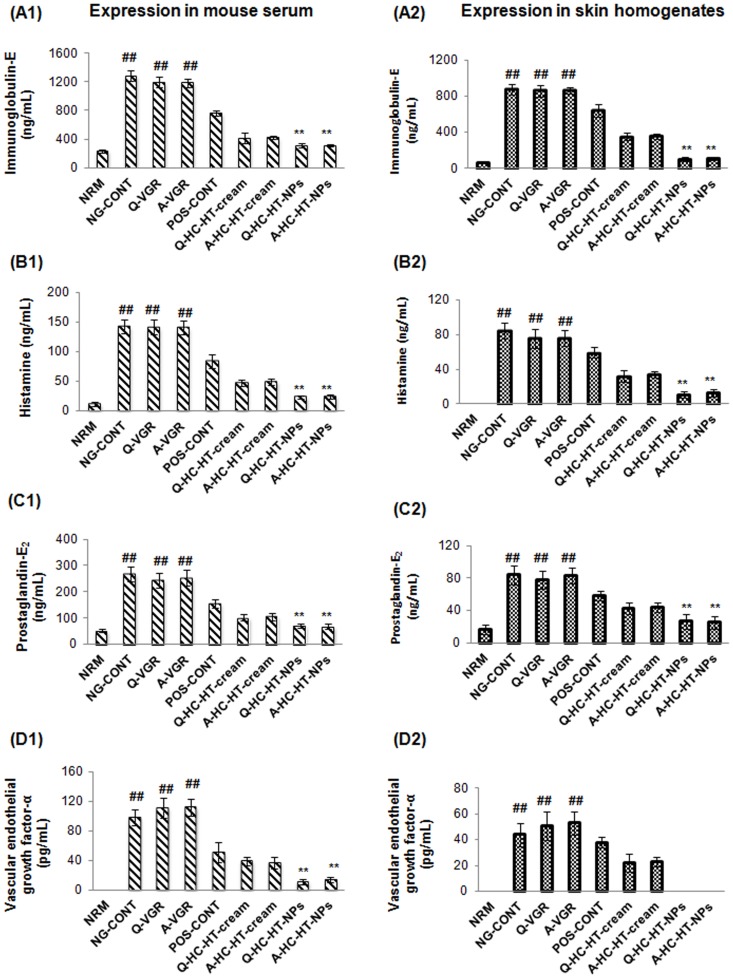
Anti-AD efficacy of NPs-based formulations. Effect of NP-based formulations on the expression of IgE, histamine, PGE_2_, and VEGF-α in serum (A1, B1, C1, and D1) and skin tissue homogenates (A2, B2, C2, and D2) of AD-induced NC/Nga mice groups compared to other groups tested. Data are presented as mean ± S.D of triplicate experiments; ^##^p<0.005 baseline vs. atopic mice, and ^**^p<0.005 NP-based formulations vs. atopic/VGRs groups. AD, atopic dermatitis; IgE, immunoglobulin-E; NPs, nanoparticles; PGE_2_, prostaglandin-E_2_; VEGF-α, vascular endothelial growth factor-α; VGRs, vehicle groups.

The untreated atopic mice group expressed the highest level of IgE in serum (1282±72 ng/mL) and skin homogenates (876±64 ng/mL) as shown in Fig. 3 [A1] and Fig. 3 [A2], respectively. These results were in accordance with previously published reports [38], [39]. They suggested that the high level of IgE measured in this group could be associated with activation of underlying inflammatory cascades in response to repetitive applications of DNFB. As a result, class switching of B-lymphocytes provokes higher expression of local and systemic IgE that leads to severe dermatosis in the atopic group. VGRs also had high levels of IgE in both samples. In contrast, commercial DermAid 0.5% cream suppressed IgE to 767±38 ng/mL and 642±74 ng/mL in serum and skin homogenates, respectively. On the other hand, co-loaded NP-based formulations demonstrated remarkable control of IgE expression (**p<0.005, one-way ANOVA), which was more prominent in the skin homogenates. The anti-IgE effect of NP-based formulations was attributable to the synergistic action of co-loaded drugs to mitigate the progression of the underlying adaptive immune response involved in AD. Furthermore, improved control of IgE expression in the skin tissues was expected to be associated with the role of CS in retaining therapeutic concentrations of both drugs in the epidermis and dermis.

### Level of histamine

Atopic mice presented a significantly (^##^p<0.005, one-way ANOVA) higher expression of histamine in serum (143±11 ng/mL) and skin tissues (84±9 ng/mL) compared with the baseline group (Fig. 3 [B1 and B2]). This can be explained by mast cells and basophils degranulation, and subsequent systemic and/or local histamine release [40]. The immune-based cross-linking of IgE with high affinity histamine receptors on mast cells and basophils results in over-activation of cells that release high levels of histamine at inflammatory sites. The resulted elevated histamine enhances the permeability of blood vessels and therefore further facilitates infiltration of guard cells into the dermis. As a result, affected mice will have severe itching/rashes episodes and thicker skin as previously explained. No reduction in histamine was observed in both samples from VGR mice. In contrast, POS-CONT mice demonstrated a significant reduction in histamine in serum (84±11 ng/mL) and skin homogenates (59±6 ng/mL). Fig. 3 [B1 and B2] also depicts that co-loaded NP-based formulations; particularly Q-HC-HT-NPs, could significantly (^**^p<0.005, one-way ANOVA) alleviate histamine level in serum (23±3 ng/mL) and skin tissue homogenates (11±4 ng/mL) compared to atopic mice.

### PGE_2_

The NG-CONT group had the highest (^##^p<0.005, one-way ANOVA) concentration of PGE_2_ in serum (286±19 ng/mL) and skin tissues (84±17 ng/mL) (Fig. 3 [C1 and C2]). This was attributed to underlying allergic and itching/rashes episodes in response to high histamine level at the site of AD-induction. Because damages to SC due to scratching would initiate the arachidonic acid pathway to produce various prostaglandins. Similarly, VGRs expressed high levels of PGE_2_ in serum and skin tissues. Meanwhile, DermAid 0.5% considerably suppressed PGE_2_ production and the effect was more prominent in serum as shown in Fig. 3. This finding could be associated with the inhibitory effect of HC on phospholipase-A_2_, which in turn blocks local/systemic prostaglandin synthesis [41]. On the other hand, co-loaded NP-based formulations (Q-HC-HT-NPs and A-HC-HT-NPs) efficiently controlled the systemic and local production of PGE_2_ as shown in Fig. 3 [C1 and C2].

### VEGF-α

Significant up-regulation of VEGF-α in serum and skin tissues were observed in AD-induced NG-CONT and VGRs groups (^##^p<0.005, one-way ANOVA) compared to the baseline group (Fig. 3 [D1 and D2]). In contrast, VEGF-α was below the detection limit (5.0 pg/mL) in the baseline group. These findings therefore suggest that VEGF-α expression is the pathological sign for severe inflammatory events. The resulting higher level of VEGF-α initiates vasculogenesis and angiogenesis. Moreover, enhanced permeability of blood vessels and infiltration of immune cells into the skin tissues might also be associated with high expression of VEGF-α. VEGF-α act as a chemoattractant for various inflammatory cells and further aggravates underlying AD-like skin lesions, which were observed in atopic and VGR groups. The topical application of NP-based formulations significantly decreased VEGF-α level in serum and skin tissues compared to non-NP–based formulations (Fig. 3[D1 and D2]). In addition, the suppressive effect of NP-based formulations on VEGF-α expression was more pronounced in skin tissues. It is assumed that this improved anti-VEGF-α effect in the skin is due to sufficient retention of drugs at the target site by CS NPs [3].

### T_H1_ cytokines

**Figure 4:**
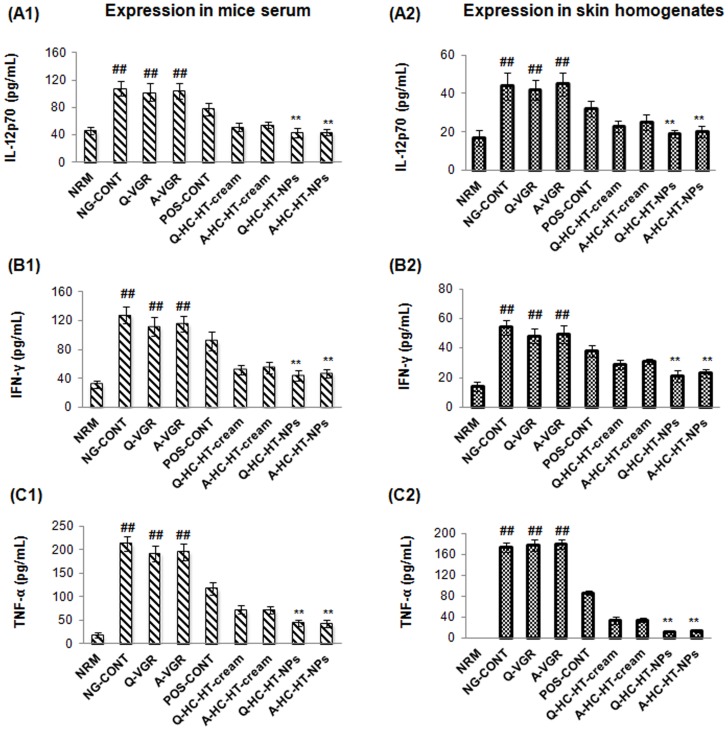
Anti-AD efficacy of NPs-based formulations. Effect of NP-based formulations on the concentration of IL-12p70, IFN-γ, and TNF-α in serum (A1, B1, and C1) and skin tissue homogenates (A2, B2, and C2) of AD-induced NC/Nga mice compared to other groups treated. Data are presented as mean ± S.D. of triplicate experiments; ^##^p<0.005 baseline vs. atopic mice, ^**^p<0.005 NP-based formulations vs. atopic/VGRs groups. AD, atopic dermatitis; IFN-γ, interferon-γ; IL-12p70, interleukin-12p70; VGRs, vehicle groups.

The therapeutic effectiveness of formulations was also explored by measuring T_H1_-specific cytokines, IL-12p70 and IFN-γ, and the pro-inflammatory cytokine, TNF-α in the present study. Fig. 4 [A1 and A2] highlights that the atopic group expressed the highest concentrations of IL-12p70 in serum (108±11 pg/mL) and skin tissues (44±9 pg/mL) respectively, compared with the baseline group (^##^p<0.005, one-way ANOVA). Similarly, Fig. 4 [B1 and B2] depicts that critically pathologic levels of IFN-γ were also measured in serum and skin homogenates of untreated AD-induced atopic mice, respectively. These findings were in agreement with previously published study [42]. According to that, IL-12p70 is over-expressed by infiltrated inflammatory cells, such as NK cells, macrophages, and eosinophils that migrate from the systemic circulation into the dermis. Overexpression of IL-12p70 mediates sequential activation of T_H0_- to T_H1_-type lymphocytes as a positive feedback mechanism. In addition, IL-12p70 stimulates signal transduction molecules to induce overproduction of other pro-inflammatory cytokines (including IFN-γ) and further aggravates underlying AD cascades. Moreover, the pleiotropic nature of IFN-γ induces proliferation and differentiation of infiltrating macrophages via macrophage-stimulating factors. Fig. 4 [C1 and C2] also highlights that the atopic mice also expressed higher TNF-α levels in serum (213±11 pg/mL) and skin tissues (174±9 pg/mL) (^##^p<0.005, one-way ANOVA) compared to the baseline group. The high expression of TNF-α could also due to higher numbers of macrophage and basophils infiltrated into the dermis.

Slight reductions in IL-12p70, IFN-γ, and TNF-α were observed in serum and skin tissue samples from VGRs. On the other hand, DermAid 0.5% considerably suppressed the expression T_H1_- and pro-inflammatory cytokines in both samples. This effect of commercial formulation was expected on the basis of HC diminished infiltration of inflammatory cells that produce 12p70, IFN-γ, and TNF-α. Contrarily, the NP-based formulations remarkably suppressed AD-responsible T_H1_- and pro-inflammatory cytokines (**p<0.005, one-way ANOVA), and reduced levels were measured in skin tissue than in serum due to the presence of CS NPs as previously discussed.

### T_H2_ cytokines

**Figure 5:**
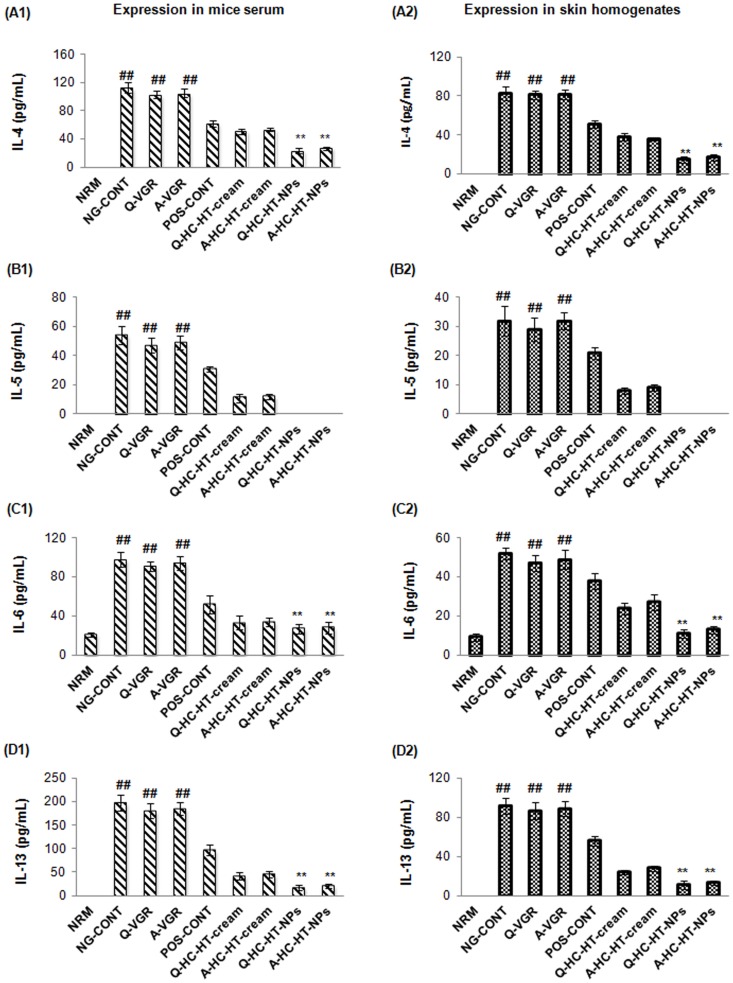
Anti-AD efficacy of NP-based formulations. Effect of NP-based formulations on the expression of IL-4, IL-5, IL-6, and IL-13 in serum (A1, B1, C1, and D1) and skin tissue homogenates (A2, B2, C2, and D2) of AD-induced NC/Nga mice compared to other groups treated. Data are presented as mean ± S.D. of triplicate experiments; ^##^p<0.005 baseline vs. atopic mice, ^**^p<0.005 NP-based formulations vs. atopic/VGRs groups. AD, atopic dermatitis; IL-, interleukin-; NPs, nanoparticles; VGRs, vehicle groups.

The T_H2_-specific cytokines, IL-4, IL-5, and IL-13, and the pro-inflammatory cytokine, IL-6 were also measured (Fig. 5) in the current study. Significantly elevated concentrations of IL-4, IL-5, IL-6, and IL-13 were observed in serum and skin tissue samples of atopic mice. This finding therefore suggests that the higher pathology of AD-like skin lesions observed in atopic mice is also associated with higher expression of T_H2_-specific cytokines and IL-6. T_H2_ cytokines further induce differentiation of T_H0_ cells into T_H2_ lymphocytes in an auto-regulatory fashion that further aggravates underlying AD reactions. Our findings are in accordance with a previous study by Suda et al. [43]. Moreover, the high expression of IgE and histamine in atopic mice could also be associated with the expression ofT_H2_-cytokines responsible for the class switching of immunoglobulin M into IgE and degranulation of mast cells and basophils to release histamine locally and/or systemically. Hence, inhibition of T_H2_- and pro-inflammatory cytokines is of prime importance to mitigate the progression of AD-like skin lesions.

Similar to other mediators, VGRs also expressed elevated T_H2_-specific and pro-inflammatory cytokines in serum and skin tissue samples. However, when AD-induced mice were treated with DermAid 0.5% cream, reductions in T_H2_-specific and pro-inflammatory cytokines were observed; lower levels were measured in serum. We also demonstrated that non-NPs–based formulations could further reduce T_H2_-specific cytokines except for IL-4. Interestingly, the co-loaded NP-based formulations; particularly Q-HC-HT-NPs, could also remarkably alleviate T_H2_-specific cytokines and the pro-inflammatory cytokine; this finding was more prominent in skin tissue as shown in Fig. 5.

### Histological examinations

#### H & E staining

**Figure 6:**
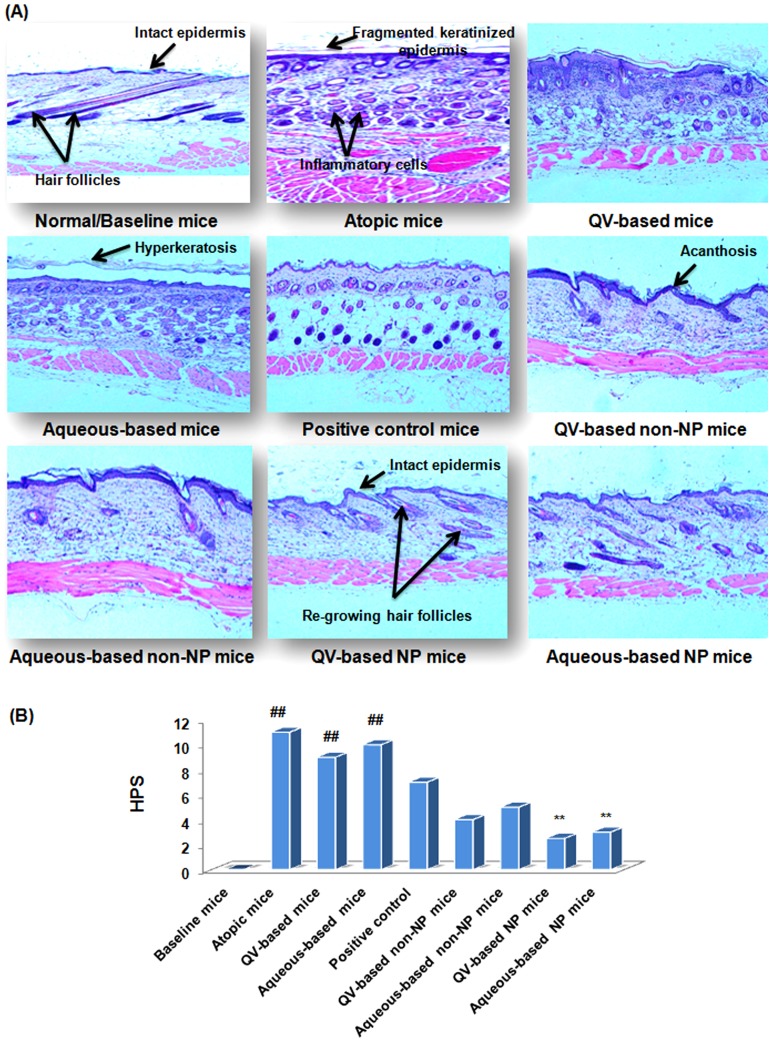
Histological stabilization of NP-based formulation. Clinical efficacy of NP-based formulations compared to normal/baseline and other treatment groups in term of histological photomicrographs (A) and HPS (B) of AD-like skin lesions of NC/Nga mice. Skin specimens stained with H & E were imaged at ×100-µm magnification. AD, atopic dermatitis; HPS, histopathological scores, H & E, hematoxylin-eosin; NPs, nanoparticles.

Fig. 6 presents photomicrographs of histological features of the integumentary system in all experimental NC/Nga mice. The histopathological severity of AD was assessed by 2 pathologists according to the following criteria: (1) Fragmentation of keratinized epithelium, (2) acanthosis, (3) number of inflammatory cells infiltrated from systemic circulation into the dermis, and (4) hyperkeratosis. Each of the criteria was scored as 0 (none), 1 (mild), 2 (moderate), or 3 (severe). The sum of the individual scores was then taken as histopathological scores (HPS) of group tested. Fig. 6 [A] depicts that AD-induced atopic mice exhibited pronounced epidermal hyperplasia, acanthosis, hyperkeratosis, fragmented keratinized epithelium, and a large number of infiltrated inflammatory cells in the papillary dermis. These pathological features were in response to the highest grades of allergic inflammatory reaction beneath the skin due to repeated applications of DNFB. Analysis of photomicrographs from atopic mice further reveals that the outer keratinized epidermal layer is separated from the inner intact epidermal layer, and this was caused by ruthless scratching of dorsal body region due to severe itching/rashes episodes. These histopathological features of atopic group caused the highest HPS of this group as shown in Fig. 6 [B]. The photomicrographs of VGRs groups show similar pathological features; however, hyperkeratosis and acanthosis were not as severe as that of NG-CONT mice, and a reduced number of infiltrated cells were observed in the dermis. In contrast, AD-induced mice treated with DermAid 0.5% presented better control of inflammatory cells infiltration and exhibited minimal epidermal hyperplasia and hyperkeratosis. Fig. 6 [A] also depicts that AD-induced mice treated with non-NPs–based formulations have shown a reduced number of infiltrated cells in the dermis and low degree of acanthosis. However, greater extent of hyperkeratosis observed in non-NP-based formulation might be the reason for more HPS (5.0), and it was expected to be due to over-hydration of the SC. On the other hand, AD-induced mice treated with NP-based formulations (HPS, 2.5 for QV-based and 3.5 for aqueous-based) show remarkable control of infiltrated cells, hyperkeratosis, acanthosis, and epidermal and dermal thickness. Moreover, HPS of QV- was lower than aqueous-based NP formulations because drug permeation from the QV-cream into the deeper skin layer was higher (Fig. S2). The higher percentage of white liquid paraffin, white soft paraffin and glycerol in QV-cream restores SC hydration that reduces dryness and itching. This, subsequently reduces scratching and SC destruction that lower the HPS. Besides, hydration of SC enhances permeation flux of the entrapped drugs. In addition, the mice treated with NP-based formulations also exhibited inter-digitations between the epidermis and dermis with greater developed hair follicles, as was observed in the baseline group. This finding indicates restoration of skin integrity in NC/Nga mice treated with NP-based formulations.

### Masson's trichrome staining

**Figure 7:**
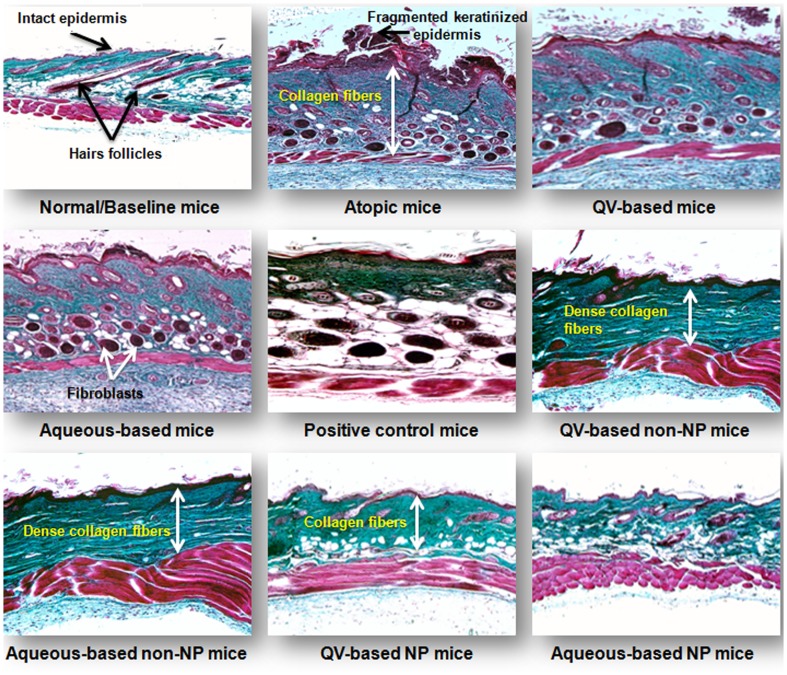
Histological stabilization of NP-based formulation. Histological photomicrographs of AD-like skin lesions of NC/Nga mice treated with NP-based formulations compared to normal/baseline mice and treatment groups. Arrows indicate the magnitude of collagen fibers deposition. Note that collagen fibers were stained green by Masson's trichrome. Photomicrographs were imaged under ×100-µm magnification. AD, atopic dermatitis; NPs, nanoparticles.

Processed skin specimens were also stained with Masson's trichrome to explore anatomical and histological changes produced in collagen fibers. Results obtained are presented in Fig. 7 as photomicrographs representing degree of collagen scaffold deposition (acquiring green color with Masson's trichrome) in the dermis. NG-CONT mice had shown highest deposition of collagen fibers in papillary and reticular layers of the dermis. Moreover, the atopic mice were also presented with the highest number of fibroblasts in the reticular dermis, with significant damage to the epidermal layers was also observed. These conditions could be explained by repeated topical applications of DNFB that led to fibrogenesis with elevated production and deposition of collagen fibers in the dermal layers. Similarly, VGRs also showed similar deposition of collagen fibers and number of fibroblast as observed in the atopic mice group. Processed skin sections of POS-CONT showed considerably lower degree of collagen fibers deposited in the papillary dermal layer because HC suppressed fibrogenesis and infiltration of fibroblasts. Mice treated with non-NPs–based formulations demonstrated greater control of fibroblast infiltration, although higher collagen fibers deposition was observed compared to POS-CONT mice as shown in Fig. 7. On the other hand, when mice were treated with co-loaded NP-based formulations, a remarkably lower degree of fibrogenesis and number of infiltrated fibroblasts was observed. The finding of a lower number of fibroblasts is expected to play a key role in reducing tissue remodeling, skin fibrosis, and scar formation secondary to AD-like skin lesions. The lowering effect of NP-based formulations might be due to synergistic actions of HC, HT, and CS to alleviate underlying inflammatory reactions involved in initiating fibrogenesis. Fig. 7 also shows a photomicrograph of baseline mice, demonstrating the presence of well-developed hair follicles and normal deposition of collagen fibers responsible for maintaining skin texture and integrity.

### VVG staining

**Figure 8:**
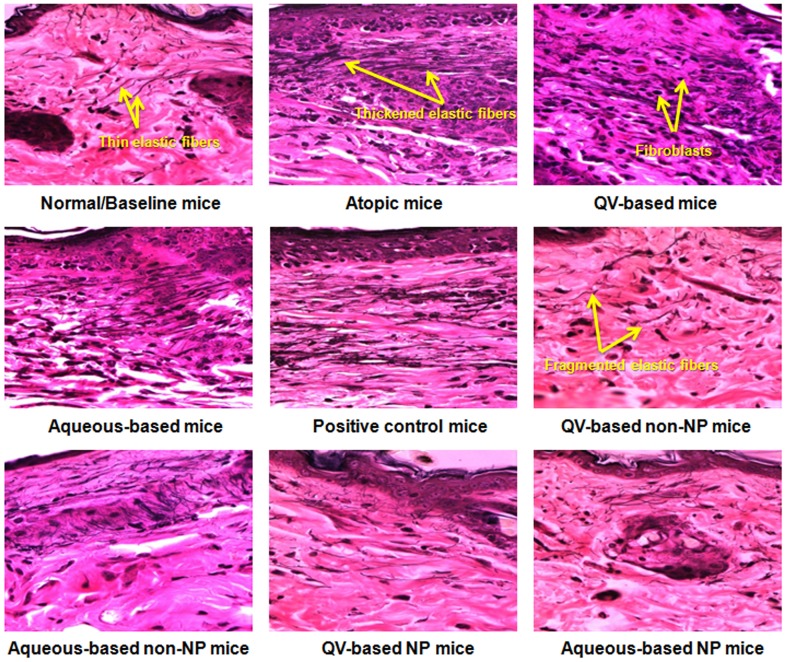
Histological stabilization of NP-based formulation. Histological photomicrographs of AD-like skin lesions of NC/Nga mice treated with NP-based formulations compared to normal/baseline mice and treatment groups. The wavy fibers, which were stained black with VVG, represent elastic fibers. Photomicrographs were imaged under ×400-µm magnification. AD, atopic dermatitis; NPs, nanoparticles; VVG, Verhoeff-Van Giesen.

In the present study, skin sections were also stained with VVG stain to examine histological changes occurring in elastic fibers (acquiring black color with VVG stains) during the course of dermatosis. The resulting photomicrographs are presented in Fig. 8. It was observed that NG-CONT mice had shown dense and thickened elastic fibers in the papillary and reticular layers of the dermis. Moreover, a high number of infiltrated fibroblasts were observed in the dermis in response to underlying inflammatory cascades of AD, which would expectedly reduce skin elasticity and resilience. The structure of these macromolecules is important for skin elasticity, and changes to these macromolecules may lead to skin fibrosis. Skin sections of VGR groups showed similar thickening of elastic tissues and fibroblasts infiltration, although the density of elastic fibers was reduced. Photomicrograph from the POS-CONT group showed a reduced number of infiltrated fibroblasts, although similar thickness of elastic fibers was observed as in the NG-CONT and VGR groups. Based on these findings, the commercial HC 0.5% formulation is thought to reduce skin elasticity and resilience. Microscopic examinations of processed skin sections treated with non-NPs–based formulations demonstrated better control of fibroblast infiltration and thickening of elastic fibers. However, elastic fibers were shortened, fragmented, and nonparallel to the epidermis; this would expectedly reduce skin elasticity. In normal anatomy and physiology, elastic fibers are arranged in parallel to the epidermis. Interestingly, the co-loaded NP-based formulations remarkably diminished fibrogenesis and thickening of elastic connective tissues. In addition, mice treated with co-loaded NP-based formulations also showed long and thin elastic fibers arranged in parallel to the epidermis like baseline group.

## Conclusions

In the present investigation, HC/HT co-loaded NPs were successfully compounded as topical creams formulations to improve the risk-benefit ratio of entrapped drugs and occlusiveness when applied to AD-like skin lesions. NP-based formulations showed pseudoplastic flow and non-Newtonian mechanics. NP-based formulations remarkably reduced the pathological severity of AD (ADI, mild) compared to atopic mice (ADI, severe). In addition, these formulations were able to reduce dorsal skin thickness in AD-induced mice. Analysis of the immuno-spectrum of AD clearly highlighted the effectiveness of NP-based formulations in alleviating the expression of IgE, histamine, PGE_2_, VEGF-α, T_H1_/T_H2_, and pro-inflammatory cytokines in serum and skin tissue homogenates. Their anti-AD efficacy was further supported by their promising potential to control pathological features, including anatomical infrastructure of skin, collagen fiber deposition, fibroblast infiltration, and thickness of elastic fibers. In conclusion, HC/HT co-loaded NP-based formulation could be used as an alternative therapeutic approach in the management of dermatosis.

## Supporting Information

Figure S1 **In-vitro release profile.** In-vitro release of HC (A) and HT (B) from lyophilised co-loaded CS NPs at pH 4.0 and 7.0 of releasing media (PBS). Results are presented as mean ± S.D (n = 3). The study was carried out using dialysis membrane bag (molecular cut-off of 12–14 kDa). The whole system was maintained at 37.0±0.5°C and stirred magnetically for 24 h. The cumulative release of HC and HT from HC/HT co-loaded NPs were measured using HPLC. The in-vitro drug release of HC/HT co-loaded CS NPs was conducted at pH 4.0 and 7.4 to mimic the pH of intact skin and inflammatory skin lesions, respectively. The data demonstrates that the co-loaded CS NPs exhibited biphasic release pattern with the initial fast release up to 12 h and subsequent slow release up to 24 h. Approximately ∼75% of the initial incorporated HC was released up to 12 h. However, after the fast initial release, the rate of drug release became slower and ∼82% of initial drug was released after 24 h. On the other hand, the higher pH (7.4) favours the release of drugs compared to the lower one (p<0.05, one way ANOVA). At pH 4.0; only ∼50% of the initial incorporated drug was released after 12 h. The release rate of HC was also slower after 12 h and the total amount released was ∼65% (Fig. S1[A]). Similar release pattern was observed for HT in which initial fast release of ∼65% of loaded drug was recorded during the first 12 h (Fig. S1[B]). After that, a slower release rate was observed and ∼70% of loaded HT was released after 24 h. The amount of HT released at pH 4.0 was ∼50% up to 12 h, followed by a slower release of ∼60% of drug was observed after 24 h.
(TIF)

Figure S2 **Ex-vivo permeation studies.** Ex-vivo Franz diffusional permeation of HC (A) and HT (B) across the full-thickness dermatomed NC/Nga mouse skin from QV- and aqueous-based HC/HT co-loaded nanoparticulate formulations compared to POS-CONT and non-NPs formulations. Data is presented as mean ± S.D, n = 3; and significance of **p<0.005 for HC/HT co-loaded NPs- formulations compared to POS-CONT and non-NPs-based formulations. Fig. S2[A] shows that a higher amount (∼32% of initial contents) of HC was measured in the receiver compartment after 24 h for POS-CONT. HC had permeated more efficiently from the QV-based non-NPs-based formulation (∼23% of initial contents) compared to the aqueous one (∼20% of initial contents). The total amount of HC permeated from POS-CONT formulation was ∼2414 µg/cm^2^ after 24 h compared to ∼1784 µg/cm^2^ and ∼1547 µg/cm^2^ for Q-HC-HT-cream and A-HC-HT-cream formulations, respectively ([A]). Moreover, the permeation coefficient (cm/h) of HC across the mouse skin was 20×10-3 cm/h, 14.9×10-3 cm/h, and 12.8×10-3 cm/h for POS-CONT, Q-HC-HT-cream, and A-HC-HT-cream formulations, respectively. The permeation of HC from co-loaded NPs-based formulations (8.2% and 7.2% for Q-HC-HT-NPs & A-HC-HT-NPs, respectively) had significantly reduced as shown in Fig. S2[A]. The total permeation of HC that had been permeated from Q-HC-HT-NPs and A-HC-HT-NPs were ∼625 and ∼595 µg/cm^2^ compared to ∼888 and ∼796 µg/cm^2^ for Q-HC-NPs and A-HC-NPs, respectively. The corresponding permeation flux (J/h) of HC across the mouse skin was ∼26 and ∼24.8 µg/cm^2^/h for Q-HC-HT-NPs and A-HC-HT-NPs, respectively. Based on the calculated value of permeability coefficient (Kp) for Q-HC-HT-NPs and A-HC-HT-NPs (5.2 and 4.9 cm/h, respectively), the co-loaded NPs-based formulations significantly (p<0.005, one-way ANOVA) reduced the rate and extent of permeation of both drugs across the mouse skin compared to the non-NPs-based formulations. Fig. S2[B] shows that ∼2727 and ∼3152 µg/cm^2^ amounts of HT had permeated across the mouse skin from Q-HC-HT-cream and A-HC-HT-cream respectively, after 24 h. The permeation flux of HT for Q-HC-HT-cream and A-HC-HT-cream formulations across the mouse skin were ∼113 and ∼131 µg/cm^2^/h, respectively. The rate and extent of HT permeation across the NC/Nga mouse skin was slightly higher in case of QV-based non-NPs formulation compared to aqueous-based non-NPs formulation. The co-loaded NPs-based formulations (Q-HC-HT-NPs and A-HC-HT-NPs) significantly (p<0.005, one-way ANOVA) reduced the permeated amount of HT (∼9% and ∼12% of the initial HT content) across the NC/Nga mouse skin. The corresponding permeation flux (J/h) of HT across the mouse skin was ∼24 and ∼32 µg/cm^2^/h for Q-HC-HT-NPs and A-HC-HT-NPs, respectively. The lower calculated values of Kp for Q-HC-HT-NPs and A-HC-HT-NPs (3.7 and 4.9 cm/h) suggested that the NPs-based formulations significantly (p<0.005, one-way ANOVA) reduced the rate and extent of HT permeation across the mouse skin compared to non-NPs-based formulations.
(TIF)
