# Supplementary figures and images for: Efficient Immuno-Modulation of TH1/TH2 Biomarkers in 2,4-Dinitrofluorobenzene-Induced Atopic Dermatitis: Nanocarrier-Mediated Transcutaneous Co-Delivery of Anti-Inflammatory and Antioxidant Drugs
Source: PLoS One. 2014 Nov 14;9(11):e113143. doi: 10.1371/journal.pone.0113143 (PMC4232601; doi:10.1371/journal.pone.0113143)

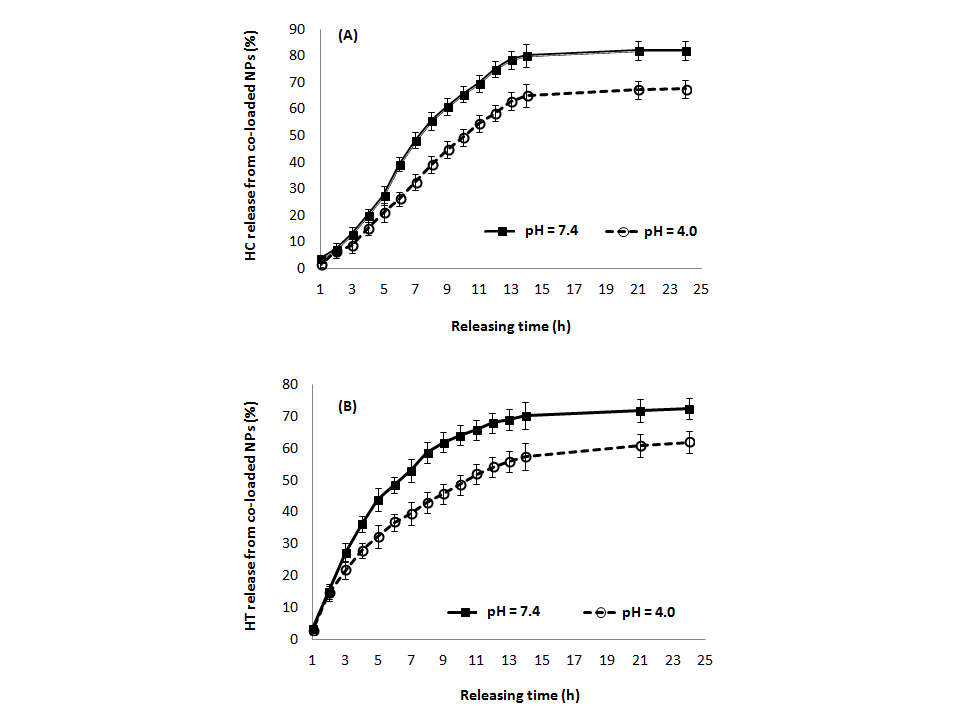

Supplement: Figure S1 — In-vitro release profile. In-vitro release of HC (A) and HT (B) from lyophilised co-loaded CS NPs at pH 4.0 and 7.0 of releasing media (PBS). Results are presented as mean ± S.D (n = 3). The study was carried out using dialysis membrane bag (molecular cut-off of 12–14 kDa). The whole system was maintained at 37.0±0.5°C and stirred magnetically for 24 h. The cumulative release of HC and HT from HC/HT co-loaded NPs were measured using HPLC. The in-vitro drug release of HC/HT co-loaded CS NPs was conducted at pH 4.0 and 7.4 to mimic the pH of intact skin and inflammatory skin lesions, respectively. The data demonstrates that the co-loaded CS NPs exhibited biphasic release pattern with the initial fast release up to 12 h and subsequent slow release up to 24 h. Approximately ∼75% of the initial incorporated HC was released up to 12 h. However, after the fast initial release, the rate of drug release became slower and ∼82% of initial drug was released after 24 h. On the other hand, the higher pH (7.4) favours the release of drugs compared to the lower one (p<0.05, one way ANOVA). At pH 4.0; only ∼50% of the initial incorporated drug was released after 12 h. The release rate of HC was also slower after 12 h and the total amount released was ∼65% (Fig. S1[A]). Similar release pattern was observed for HT in which initial fast release of ∼65% of loaded drug was recorded during the first 12 h (Fig. S1[B]). After that, a slower release rate was observed and ∼70% of loaded HT was released after 24 h. The amount of HT released at pH 4.0 was ∼50% up to 12 h, followed by a slower release of ∼60% of drug was observed after 24 h. (TIF) [file pone.0113143.s001.tif]

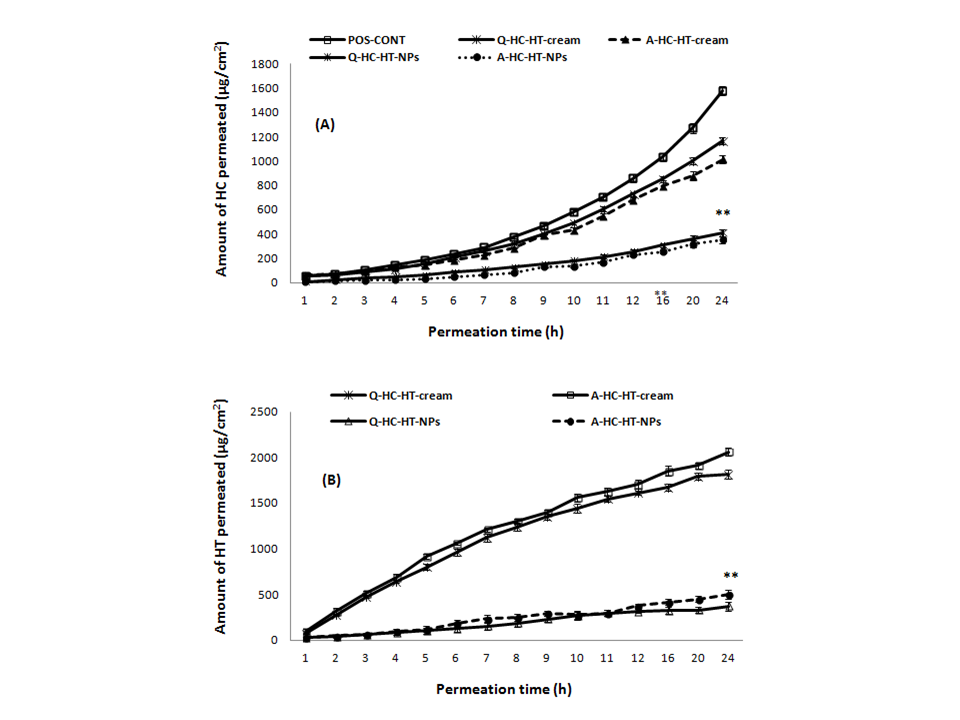

Supplement: Figure S2 — Ex-vivo permeation studies. Ex-vivo Franz diffusional permeation of HC (A) and HT (B) across the full-thickness dermatomed NC/Nga mouse skin from QV- and aqueous-based HC/HT co-loaded nanoparticulate formulations compared to POS-CONT and non-NPs formulations. Data is presented as mean ± S.D, n = 3; and significance of **p<0.005 for HC/HT co-loaded NPs- formulations compared to POS-CONT and non-NPs-based formulations. Fig. S2[A] shows that a higher amount (∼32% of initial contents) of HC was measured in the receiver compartment after 24 h for POS-CONT. HC had permeated more efficiently from the QV-based non-NPs-based formulation (∼23% of initial contents) compared to the aqueous one (∼20% of initial contents). The total amount of HC permeated from POS-CONT formulation was ∼2414 µg/cm2 after 24 h compared to ∼1784 µg/cm2 and ∼1547 µg/cm2 for Q-HC-HT-cream and A-HC-HT-cream formulations, respectively ([A]). Moreover, the permeation coefficient (cm/h) of HC across the mouse skin was 20×10-3 cm/h, 14.9×10-3 cm/h, and 12.8×10-3 cm/h for POS-CONT, Q-HC-HT-cream, and A-HC-HT-cream formulations, respectively. The permeation of HC from co-loaded NPs-based formulations (8.2% and 7.2% for Q-HC-HT-NPs & A-HC-HT-NPs, respectively) had significantly reduced as shown in Fig. S2[A]. The total permeation of HC that had been permeated from Q-HC-HT-NPs and A-HC-HT-NPs were ∼625 and ∼595 µg/cm2 compared to ∼888 and ∼796 µg/cm2 for Q-HC-NPs and A-HC-NPs, respectively. The corresponding permeation flux (J/h) of HC across the mouse skin was ∼26 and ∼24.8 µg/cm2/h for Q-HC-HT-NPs and A-HC-HT-NPs, respectively. Based on the calculated value of permeability coefficient (Kp) for Q-HC-HT-NPs and A-HC-HT-NPs (5.2 and 4.9 cm/h, respectively), the co-loaded NPs-based formulations significantly (p<0.005, one-way ANOVA) reduced the rate and extent of permeation of both drugs across the mouse skin compared to the non-NPs-based formulations. Fig. S2[B] shows that ∼2727 and ∼3152 µg/cm2 amounts of HT h [file pone.0113143.s002.tif]
